# Pharmacokinetic Interactions for Drugs with a Long Half-Life—Evidence for the Need of Model-Based Analysis

**DOI:** 10.1208/s12248-015-9829-2

**Published:** 2015-10-13

**Authors:** Elin M. Svensson, Chayan Acharya, Björn Clauson, Kelly E. Dooley, Mats O. Karlsson

**Affiliations:** 1 Department of Pharmaceutical Biosciences, Uppsala University, P.O. Box 591, 751 24 Uppsala, Sweden; 2 Department of Medicine, Johns Hopkins University School of Medicine, Baltimore, Maryland USA

**Keywords:** drug-drug interactions, long half-life, model-based analysis, non-compartmental analysis, pharmacokinetics

## Abstract

Pharmacokinetic drug-drug interactions (DDIs) can lead to undesired drug exposure, resulting in insufficient efficacy or aggravated toxicity. Accurate quantification of DDIs is therefore crucial but may be difficult when full concentration-time profiles are problematic to obtain. We have compared non-compartmental analysis (NCA) and model-based predictions of DDIs for long half-life drugs by conducting simulation studies and reviewing published trials, using antituberculosis drug bedaquiline (BDQ) as a model compound. Furthermore, different DDI study designs were evaluated. A sequential design mimicking conducted trials and a population pharmacokinetic (PK) model of BDQ and the M2 metabolite were utilized in the simulations where five interaction scenarios from strong inhibition (clearance fivefold decreased) to strong induction (clearance fivefold increased) were evaluated. In trial simulations, NCA systematically under-predicted the DDIs’ impact. The bias in average exposure was 29–96% for BDQ and 20–677% for M2. The model-based analysis generated unbiased predictions, and simultaneous fitting of metabolite data increased precision in DDI predictions. The discrepancy between the methods was also apparent for conducted trials, *e.g.*, lopinavir/ritonavir was predicted to increased BDQ exposure 22% by NCA and 188% by model-based methods. In the design evaluation, studies with parallel designs were considered and shown to generally be inferior to sequential/cross-over designs. However, in the case of low inter-individual variability and no informative metabolite data, a prolonged parallel design could be favored. Model-based analysis for DDI assessments is preferable over NCA for victim drugs with a long half-life and should always be used when incomplete concentration-time profiles are part of the analysis.

**Electronic supplementary material:**

The online version of this article (doi:10.1208/s12248-015-9829-2) contains supplementary material, which is available to authorized users.

## INTRODUCTION

Simultaneous administration of multiple drugs is a common practice in current medicine, for instance, in the case of polypharmacy among the elderly or in the treatment of infectious diseases such as HIV and/or tuberculosis, each of which require a combination therapy with three or more compounds to achieve stable cure and avoid the emergence of resistance. When multiple drugs are administered simultaneously, clinically important drug-drug interactions (DDIs) may occur (1–4). Pharmacokinetic (PK) DDIs, which are the focus of this study, can result in undesirably low or high levels of drug exposure yielding the treatment either inefficacious or toxic. An accurate estimate of the impact of the DDI on drug exposures is therefore essential to first of all assess the need for dose adjustment and then to make dose adjustment recommendations.

PK DDIs are traditionally evaluated in single-dose studies with cross-over or sequential designs where the PK of the victim drug (the drug of primary interest) after administration with and without the perpetrator drug (the drug potentially impacting the victim drug) are compared, as described in the regulatory guidelines from EMA and FDA (5,6). PK parameters of interest (area under the concentration curve [AUC] and *C*_max_) are commonly derived with non-compartmental analysis (NCA), and the DDI is expressed as a geometric mean ratio (GMR) of these parameters. However, this approach becomes problematic when the elimination half-life of the drug of interest is exceedingly long since this may lead to infeasibly long wash-out periods, carry-over between the dosing occasions, impractical and expensive long sampling periods, and/or incomplete capturing of the full concentration-time profile.

Bedaquiline (BDQ) is a new antituberculosis drug with multi-phasic elimination and a terminal elimination half-life of more than 5 months (7). BDQ is mainly metabolized through N-demethylation catalyzed by the cytochrome P450 3A4 isoenzyme (CYP3A4) forming the metabolite M2, which in turn can be metabolized by the same process into M3 (7). M2 is less active than BDQ but has been linked to potential safety concerns (7). Urinary excretion of BDQ is negligible; fecal excretion occurs but the extent is unknown (7,8).

Since tuberculosis is always treated with a combination therapy regimen and a large part of patients receive concomitant antiretroviral treatment due to HIV co-infection (9) (*e.g.*, 32% of the 1.1 million TB patients with HIV co-infection were started on antiretroviral therapy globally in 2013 and the number is increasing (10)), correct assessment of DDIs is essential for efficient and safe application of BDQ. A number of DDI studies have been conducted and analyzed with both traditional NCA and model-based population PK methods, with the results differing substantially between the two methods for some studies (11–16). The objective of the current study was to compare NCA and model-based predictions of DDIs in conducted trials with BDQ and in a simulation study with BDQ as a model for long half-life drugs and to investigate how various study designs influence the bias and precision of DDI assessments. This study will provide general recommendations for study design and analysis methods to generate accurate predictions of DDIs involving a compound with long half-life to support future regulatory guidance.

## METHODS

### Population PK Model

A previously published population model describing the PK of BDQ and M2 was used as the basis for the simulation study (14). The model was developed using data from a phase I DDI study with sequential design including 35 subjects investigating the influence of efavirenz on BDQ and M2 PK. Plasma concentrations for PK analysis were measured over 2 weeks after a single 400-mg BDQ dose was administrated, which was followed by a wash-out period of 2 weeks when daily administration of 600 mg efavirenz was initiated and maintained throughout the study. Four weeks after the first dose, a second 400-mg BDQ dose was administered (together with efavirenz), and BDQ and M2 concentrations were again measured over the following 2 weeks. Each observation period included one pre-dose sample and 16 samples between 1 and 336 h post-dose with closer observations in the early part of the period. The developed population model included a dynamic transit-compartment structure describing the absorption and three and two compartments for the disposition of BDQ and M2, respectively. The bioavailability and fraction BDQ metabolized to M2 were set to 1 in the model; hence, estimated parameters are relative to the bioavailability and for the metabolite also to the fraction BDQ metabolized to M2 (fm). Allometric scaling with body weight was included in the model. The model was implemented in NONMEM 7.3 (17) and utilized the first-order conditional estimation method with interaction (FOCEI). Supplementary Material 1 includes a schematic figure of the model structure and model parameter estimates.

### Posterior Predictive Check

The suitability of the model and study design for the simulations mimicking DDI studies was evaluated with posterior predictive check (PPC) methodology (18). Secondary PK parameters estimated with NCA using the originally observed data were compared with the same parameters estimated from a large number (*n* = 1000) of datasets simulated with the model. The study design, PK sampling schedule, and the weight characteristics of the subjects were the same in the simulations as in the original study. The R-package *ncappc* (19–21) and the *nca* functionality in PsN were utilized for this task (22,23). The parameter of main interest was GMR based on AUC_0–336 h_. In addition to the study of DDI with efavirenz, the same evaluation was conducted on models with the same structure describing the DDIs with nevirapine, ritonavir-boosted lopinavir, rifampicin, and rifapentine (15,16). The previously performed clinical studies were conducted in accordance with GCP and local ethical guidelines.

### Simulation Study of DDI Predictions

**Fig. 1:**
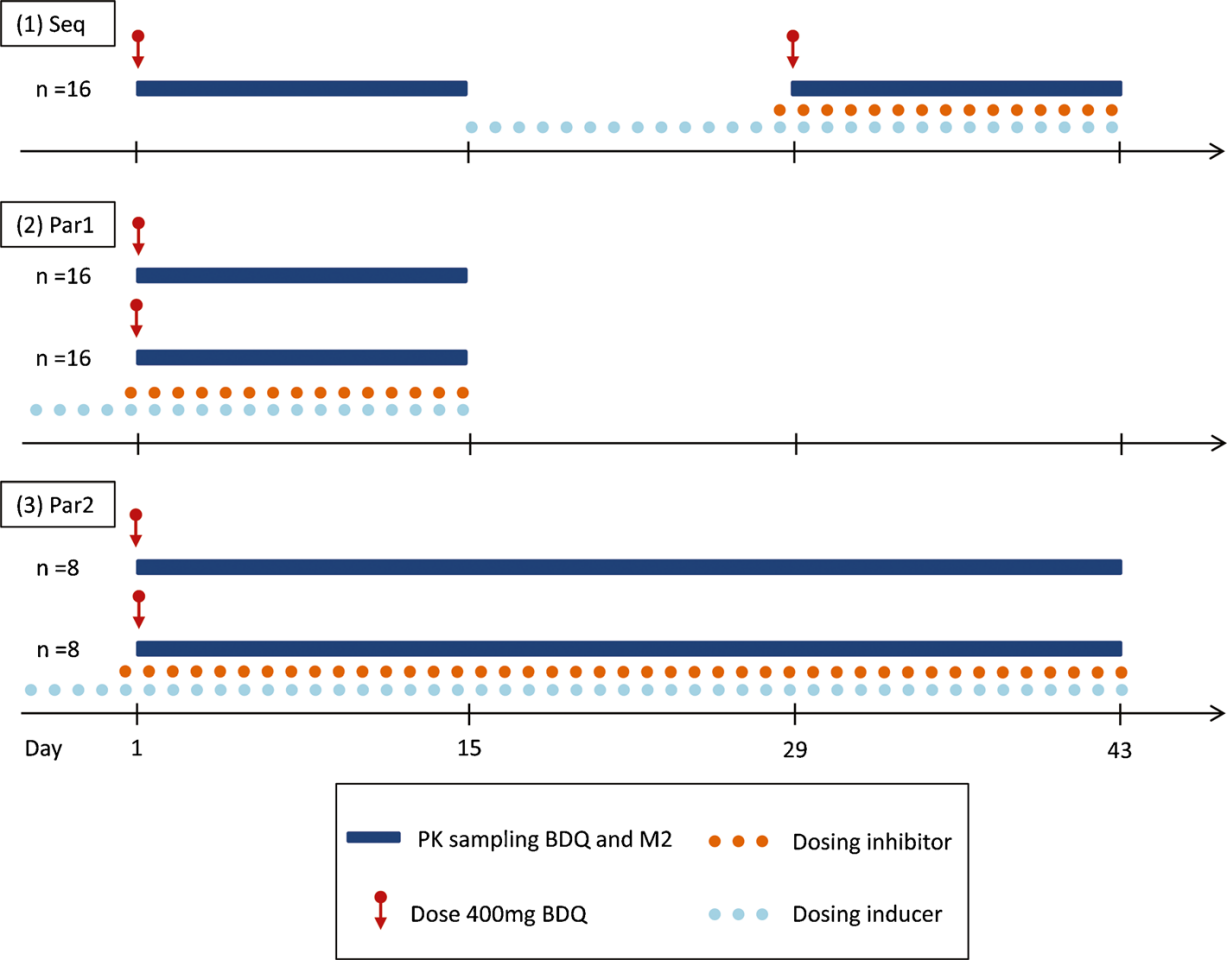
Schematic illustration of study designs (*Seq* sequential, *Par1* parallel 1, *Par2* parallel 2) evaluated for prediction of DDIs for a drug with a long half-life

The same study design and sampling strategy as in the original study were used (see original publication for details (11) and the sequential design in Fig. 1 for an overview). The number of subjects and their weight characteristics were also unaltered. Five hypothetical but realistic DDI scenarios were chosen: inhibition of BDQ clearance (CL) to 20 or 50% of normal, no interaction effect, and induction of BBQ CL to 200 or 500% of normal. The interaction effect on M2 CL was set to the same magnitude as on BDQ CL. Inter-individual variability (IIV) in interaction effect on BDQ and M2 was 20–30% and the correlation was 75%, consistent with the earlier estimates for the effect of efavirenz (14). The interaction effects were set to have full impact on CL from 1 week before the second BDQ dose, corresponding to the administration of an inhibitor starting that same day or an inducer about 1 week earlier. One hundred trials for each scenario were simulated, and the data were analyzed with NCA and by re-estimation of all model parameters, including two separate parameters for the interaction effects on BDQ and M2. Prediction of the impact of the interaction by NCA was defined as the GMR of AUC_0–336 h_ and for the model-based method as the relative average concentration at steady state (rel*C*_avg,ss_, identical to the ratio of weekly AUC at steady state with and without interaction effect) calculated from estimated apparent CLs with and without interaction effect (IE) (Eq. 1).

1 $$ \mathrm{r}\mathrm{e}\mathrm{l}{C}_{\mathrm{avg},\mathrm{s}\mathrm{s}}=\frac{C_{\mathrm{avg},\mathrm{s}\mathrm{s}}^{\mathrm{IE}}}{C_{\mathrm{avg},\mathrm{s}\mathrm{s}}}=\frac{C{L}_{\mathrm{apparent}}}{CL_{\mathrm{apparent}}^{\mathrm{IE}}} $$

The estimated CL_apparent_ corresponds to CL/F for BDQ and CL_(M2)_/(F * f_*m*_) for M2. The predictions from the two methods were compared with the true rel*C*_avg,ss_ for BDQ and M2 under the given DDI scenario and bias (reported as %) was calculated as the difference between predicted and the true relative to the true.

### Use of Metabolite Data

For the original BDQ and efavirenz study and for each of the simulated scenarios described above, an alternative model describing only BDQ PK was estimated excluding the metabolite data. The precision (relative standard error, RSE) in the parameter describing the interaction effect on BDQ for the models including both BDQ and M2 data and the models including BDQ data only were compared.

### Alternative Study Designs

Three study designs were investigated (Fig. 1).

1. A sequential design including 16 subjects with two BDQ doses administered 4 weeks apart; the second dose was administered preceded by and together with the interacting drug, 2 weeks of PK sampling after each dose (similar to the design used in conducted BDQ DDI studies).
2. A parallel design including 32 individuals, 16 in each of the two groups, where a single BDQ dose was administered with or without interacting drug and PK sampling over 2 weeks.
3. A parallel design including 16 individuals, 8 in each of the two groups, where a single BDQ dose was administered with or without interacting drug and PK sampling over 6 weeks.
  All three designs included an equal number of samples. The groups in the parallel designs were matched on body weight. Three DDI scenarios were chosen: inhibition of BDQ CL to 20% of normal, no interaction effect, and induction of BDQ CL to 500% of normal. For each DDI scenario, three different PK scenarios were evaluated: (i) IIV in BDQ and M2 clearances and interaction effects as estimated originally (24, 19, 21, and 28% coefficient of variation [CV], respectively) (called “Original”); (ii) 50% CV in BDQ and M2 clearances and IIV in the interaction effects on BDQ and M2 as estimated originally (called “High CL IIV”); and (ii) IIV of BDQ and M2 clearances as estimated originally and 50% CV in the interaction effects on BDQ and M2 (called “High IE IIV”). Correlation structures and magnitudes mimicked what has earlier been estimated for induction and inhibition effects, respectively. One hundred trials for each scenario (*n* = 9) were simulated and analyzed with NCA and model-based re-estimation of all parameters. In the NCA analysis, GMRs were calculated from both AUC_0–336 h_ and AUC_0–inf_ including an extrapolated area calculated from the last observation and the estimated terminal half-life. The terminal half-life was estimated from the slope of a regression line fitted to *n* last observations where the number *n* was determined by the adjusted regression coefficient. The re-estimation was conducted both including all data (two separate parameters for interaction effect on BDQ and M2) and including only the BDQ data.

## RESULTS

### Posterior Predictive Check

The results from the PPC are summarized in Table I. The GMRs calculated by NCA on the observed data generally agree well with the median of the GMRs calculated on simulated data and falls within the 95% confidence intervals for all five evaluated DDIs and both BDQ and M2. Hence, the model is able to generate data in good agreement with the observed data and is suitable for use in simulation studies.

**Table I:** Summary of Results from Posterior Predictive Checks Comparing GMRs Calculated on NCA Derived AUC_0–336 h_ for BDQ and M2, Respectively, with Different Perpetrator Dugs

Victim	Perpetrator	GMR of AUC_0–336 h_ original data	GMR of AUC_0–336 h_ simulated data median (95% CI)
BDQ	Efavirenz	0.868	0.785 (0.699, 0.885)
	Lopinavir/ritonavir	1.214	1.303 (1.171, 1.45)
	Nevirapine	1.028	1.164 (1.009, 1.353)
	Rifampicin	0.41	0.406 (0.345, 0.477)
	Rifapentine	0.428	0.478 (0.419, 0.543)
M2	Efavirenz	1.278	1.202 (1.072, 1.34)
	Lopinavir/ritonavir	0.627	0.642 (0.552, 0.747)
	Nevirapine	1.049	1.143 (1.014, 1.313)
	Rifampicin	0.789	0.767 (0.647, 0.907)
	Rifapentine	0.855	0.89 (0.764, 1.046)

*GMR* geometric mean ratios, *AUC*_*0–336 h*_ area under the concentration-time curve between 0 and 336 h after dose, *BDQ* bedaquiline, *M2* monodesmethyl-metabolite of BDQ

### Simulation Study of DDI Predictions

**Fig. 2:**
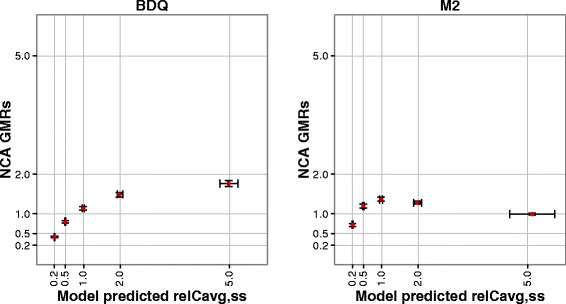
Comparison of DDI predictions from NCA (*y*-axis) and model-based analysis (*x*-axis) for the five simulated scenarios. The *gray lines* represent the true relative average steady-state concentrations (rel*C*_avg,ss_) for each scenario. The results are presented as median and inter-quartile range

Figure 2 illustrates the DDI predictions by NCA and model-based estimation, for BDQ and M2, respectively, in relation to the true Rel*C*_avg,ss_ for the given magnitude of the DDI applied in the simulation. For both induction and inhibition, the magnitude of the DDIs’ impact is under-predicted by NCA GMRs; more severely so for the metabolite. The bias was 29–96 and 20–677% for BDQ and M2, respectively, in the different scenarios. The model-based estimation accurately predicted the DDIs’ impact for both BDQ and M2 (bias was 0.1–1.1 and 1.1–4.9%, respectively), but with lower precision for the strong inhibition.

### Use of Metabolite Data

**Fig. 3:**
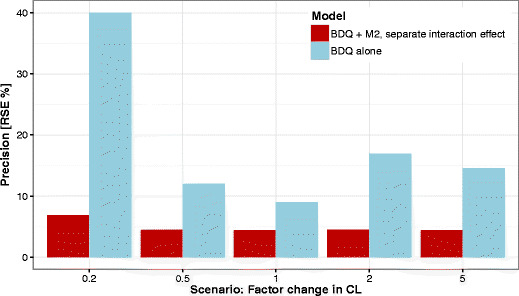
Precision of model-based DDI predictions quantified as relative standard error (RSE) of the estimate of the parameter describing DDI effect on BDQ from the 100 simulated trials for each scenario when including both BDQ and M2 data (*red bars*) or BDQ data only (*blue bars*)

The precision in the estimated DDI parameter was markedly better when both parent and the metabolite data were used in the estimation (Fig. 3). In the simulation study, RSEs were between two and six times higher when the information from the metabolite data was excluded. For the observed data, the RSE was increased fivefold.

### Alternative Study Designs

**Fig. 4:**
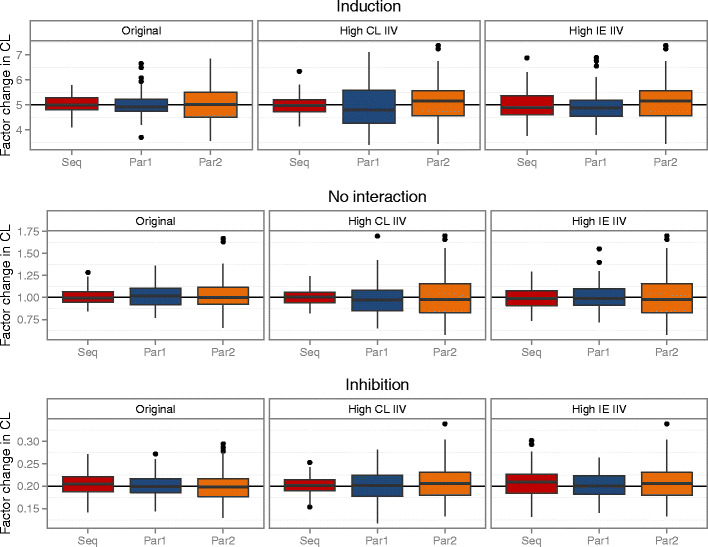
Box plots of model-based estimation of interaction effect (factor change in CL) for the different designs (*Seq* sequential, *Par1* parallel 1, *Par2* parallel 2), the different PK scenarios (original, high CL IIV, and high IE IIV), and the different interaction effect scenarios (induction, no interaction, and inhibition)

**Fig. 5:**
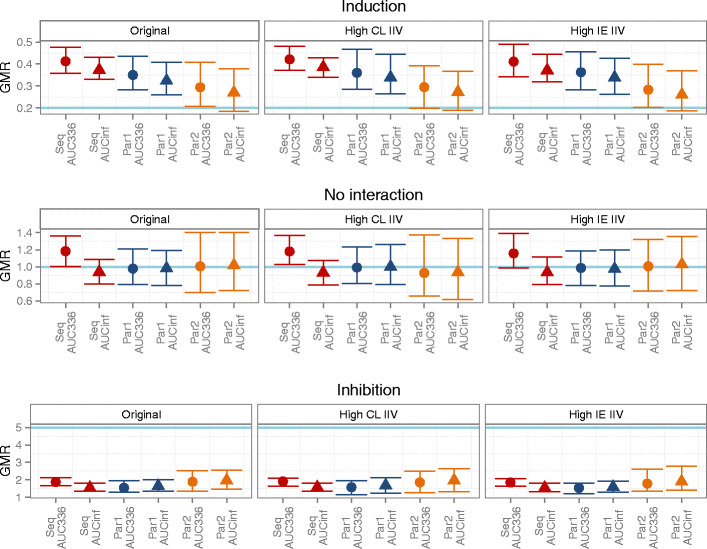
Median and 90% non-parametric CI for NCA-derived GMRs for the different designs (*Seq* sequential, *Par1* parallel 1, *Par2* parallel 2), the different PK scenarios (original, high CL IIV, and high IE IIV), and the different interaction effect scenarios (induction, no interaction, and inhibition). True impact of the simulated DDI shown as the *light blue line*

The model-based analysis generated accurate predictions of the interaction effect for all study designs and under all scenarios (Fig. 4). Generally, the sequential design resulted in the best precision with a larger gain in the scenario with higher CL IIV but little or no gain in the scenario with high interaction effect IIV. When parallel design was used, the precision was generally better in the design with standard length of the sampling period but more subjects (parallel 1) compared to the design with standard number of subjects but longer sampling period (parallel 2). However, when the estimation was conducted without the metabolite data, the parallel design with longer sampling period (parallel 2) generally performed best of all three designs, indicating the benefit of longer sampling to accurately estimate the BDQ clearance in the absence of metabolite data (Supplementary Material 2). The NCA-derived GMRs based on either AUC_0–336 h_ or AUC_0–inf_ failed to reflect the true impact of the simulated induction and inhibition in each PK scenario (Fig. 5). AUC_0–inf_ generally came closer to the true value although the uncertainty of the extrapolation was large. The problem caused by carry-over between the doses was evident for the sequential design in the case of no interaction and GMR based on AUC_0–336 h_. The parallel design with long sampling period came closer to the true values than the parallel design with increased number of subjects; however, it was less precise. The level of IIV of clearances or interaction effect had little impact on the NCA results.

## DISCUSSION

The conducted simulation study clearly demonstrates that NCA-derived GMRs underestimate the full impact of a DDI for a victim drug with long half-life (Fig. 2). The predictions are even more biased for the metabolite compared to the parent compound. The reasons are several and will be discussed in detail below. Firstly, the whole concentration-time curve could not be characterized. For BDQ, the 2 weeks of observations after each dose were not enough to observe the major part of the total AUC. Table II summarizes the estimates of the fraction observed based on model-derived AUC_0–336 h_ and AUC_0–inf_. In the absence of any interaction effect, about half of the total AUC was observed for BDQ and less than a third for M2. Hence, a substantial part of the elimination phase was ignored when GMRs based on AUC_0–336 h_ were used to predict the impact of a DDI, consequently resulting in the under-prediction of any DDI affecting the elimination. Furthermore, the fraction of the total AUC that can be observed during a fixed time period changes with the interaction effect. The GMRs will therefore compare a smaller with a larger fraction of the total AUC or *vice versa*. For example, in the case of inhibition when BDQ CL is decreased to half of its normal value, 31% of total AUC would be observed over 2 weeks and compared with 48% of total AUC observed over the same time period without interaction effect. This will further bias the prediction, aggravate the under-prediction in the case of inhibition, and somewhat counteract the under-prediction in the case of induction. Calculation of GMRs based on NCA-derived AUC_0–inf_ did unfortunately not improve the predictions substantially either. To estimate a terminal half-life known to be longer than 5 months on 2 weeks observations after a single dose using NCA is bound to be extremely uncertain. In the simulation examples, the NCA estimates of terminal half-life were around a few hundred hours, which is about ten times lower than the true value, resulting in incorrect extrapolation and little improvements in the GMRs. The predictions for the metabolite suffer from the same limitations as for the parent drug and, in addition, the added complexity that both input rate and elimination are affected. For induction affecting clearance of both parent and metabolite, more metabolite appears early during the observation period. It may therefore seem as exposure is increasing (as it does in the simulated scenario where CL of BDQ and M2 is increased to the double, see Fig. 2), despite that the true total exposure would be half of the normal exposure in the absence of any interaction effect.

**Table II:** Summary of Observed Fraction (%) of Total AUC of BDQ and M2 Observed During a 2-Week Sampling Period for the Different Interaction Scenarios

	Interaction effect (factor change in CL)	BDQ AUC_0–336 h_/AUC_0–inf_ (%)	M2 AUC_0–336 h_/AUC_0–inf_ (%)
Inhibition	0.2	15	3
	0.5	31	12
No interaction	1	48	29
Induction	2	65	54
	5	83	81

*CL* clearance, *BDQ* bedaquiline, *AUC* area under the concentration curve,M2 monodesmethyl-metabolite of BDQ

The model-based analysis accurately predicted DDI effects using a model parameterized as the parent being fully metabolized through the pathway affected by the interaction. Although the model is formulated under this assumption, it is valid for any fraction of the parent CL being induced or inhibited and will correctly predict the changes in exposure since that is determined by the total CL which is captured by the model. However, if the fraction of the parent metabolized through the induced/inhibited pathway is less than one, the interpretation of the estimated interaction effect on CL (IE_apparent_) does not reflect the change in the pathway associated with the interaction. Rather, the fractional change in the associated pathway (IE_specific_) can be obtained from Eq. 2, where fm is the fraction metabolized through the pathway in question in the absence of any interaction effect.

2 $$ {\mathrm{IE}}_{\mathrm{specific}}=\frac{{\mathrm{IE}}_{\mathrm{apparent}}+\mathrm{fm}-1}{\mathrm{fm}} $$

For inhibitors, some conclusions can often be drawn about the importance of the pathway in question from the value of the IE_apparent_. For the metabolite, the implemented model assumes that all eliminated parent is forming the measured metabolite and that all metabolite are eliminated through a pathway affected by the interaction. As for the parent compound, the model will correctly predict change in the exposure of the metabolite even if either or both of these assumptions are violated, but again, the interpretation of the estimated parameters will change. Additional discussion about the interpretation of the metabolite’s parameters and the relative magnitudes of BDQ’s and M2’s clearance pathways given the observed results in published DDI studies can be found in Supplementary Material 3.

An awareness of the poorly predicted terminal half-life and a general reluctance to use AUC_0–inf_ when the extrapolated area forms a large part of the total area are probably the reasons why all published NCA predictions of DDIs with BDQ and M2 have used AUC_0–336 h_ (7,11,12). The summary in Table III compares NCA and model-based DDI predictions and shows that NCA consistently predicts lower impact of the DDI than model-based analysis. The largest discrepancy is observed for the strong inhibitors lopinavir/ritonavir where NCA predicts a 22% increase in BDQ exposure while model-based analysis predicts an increase of 188%. These two predictions may produce a different outcome in a discussion about the clinical importance of the DDI and the need for a dose adjustment. It is also striking that NCA predicts M2 exposure to decrease more than 40% during co-administration with lopinavir/ritonavir, although inhibition of CYP3A4 is expected to affect M2 clearance and exposure therefore should increase. Recently conducted DDI studies in patients on long-term BDQ treatment with concomitant antiretroviral are expected to provide further insight in the predictive accuracy of these two methods. Preliminary results show a twofold increase in BDQ exposure when administered together with lopinavir/ritonavir but no significant change in M2 exposure (24). However, the interpretation of the presented results is difficult since sampling was conducted somewhere between week 3 and 24 of BDQ treatment, and it is expected that accumulation over time of treatment would make average concentrations of BDQ and M2 over that time period vary substantially (15).

**Table III:** Comparison of Previous Published NCA and Model-Based DDI Predictions for BDQ and M2

Victim	Perpetrator	NCA prediction^*a*^	Model-based prediction^*b*^
BDQ	Efavirenz	−18%^*c*^ (10)	−52% (13)
	Nevirapine	No change (7)	+9% (14)
	Rifampicin	−59% (11)	−79% (15)
	Rifapentine	−57% (11)	−75% (15)
	Lopinavir/ritonavir	+22% (7)	+188% (14)
M2	Efavirenz	+7%^*c*,*d*^ (10)	−52% (13)
	Nevirapine	No change (7)	−5% (14)
	Lopinavir/ritonavir	−41% (7)	+73% (14)

*NCA* non-compartmental analysis, *BDQ* bedaquiline, *AUC* area under the concentration curve, M2 monodesmethyl-metabolite of BDQ

^*a*^Ratio of AUC_0–336 h_

^*b*^Relative average concentration at steady state (equal to ratio of weekly AUC at steady state)

^*c*^Observations corrected for carry-over

^*d*^Not significantly different from 0%

BDQ which was used as an example in this work has indeed extreme PK characteristics, and the results presented here may represent one of the worst-case scenarios. However, there are numerous other drugs with very long half-life, examples are mefloquine 14–41 days (25), amiodarone 21–78 days (26), and oritavancin 393 h (27). Furthermore, what can be called “long half-life” is always relative to the length of the sampling period. In a recent DDI study, conducted in healthy volunteers, investigating the antimalarial drugs cipargamin and piperaquine (terminal half-life ~22 days) PK samples had to be collected over 60 days after a single dose which is impractical but necessary since NCA was used for the interpretation (28). The problems presented for the BDQ example will occur for any drug in any situation where it is impossible or undesirable to sample long enough to observe the majority of the total AUC. Our conclusions regarding NCA’s inability to generate accurate predictions are therefore more widely applicable and should be considered at least for any drug with half-life longer than the intended sampling period.

Measuring metabolite concentrations contributes to the ability to characterize the clearance of the parent compound in a model-based analysis since the formation of the metabolite is linked to the clearance of the parent. Thereby metabolite observations also contribute to the characterization of DDIs as shown by the increased RSE in the interaction effect parameter in our simulations when metabolite data was excluded (Fig. 3). There are also examples in the literature where simultaneous modeling of parent and metabolite data has aided characterization of DDIs, *e.g.*, the effect of ketoconazole and rifampin on ifosfamide, the effect of zosuquidar trihydrochloride on doxorubicin, or the effect of ritonavir on nelfinavir (29–31). We suggest that it could be of interest to measure metabolite concentrations, when a metabolite with relevant exposure levels exists, even in situations where the metabolite is not active or important for safety reasons. This is expected to increase the precision in the predictions which means that a study with a smaller sample size may be sufficient to predict the impact of the interaction with retained power, which in turn is beneficial from both ethical and economic perspectives.

Parallel study designs instead of cross-over or sequential designs have been suggested as options for drugs with long half-life in order to avoid carry-over. Our simulations demonstrate that overcoming the problem of carry-over still does not render NCA-derived predictions accurate (Supplementary Material 2); however, longer sampling periods do bring the predictions closer to the truth. A parallel design with longer sampling could therefore be preferred over a cross-over/sequential if NCA methodology has to be used and could potentially generate accurate predictions provided that a high enough percentage of the total AUC is observed and/or for weaker interaction effects. Correcting the AUC observed on the second occasion in a cross-over/sequential study for the carry-over by subtracting an extrapolated AUC is problematic since the terminal half-life is poorly estimated and the correction may not be reliable. For the model-based analysis, where carry-over does not necessarily introduce bias, the study design does not impact the accuracy and the precision of the DDI prediction substantially when the IIV of the clearance of the victim drug is relatively low (Fig. 4). However, when the IIV of the clearance of the victim drug is as high as 50% CV, precision is lost in the parallel designs. We therefore support the continued used of cross-over or sequential study designs when the study is intended for model-based analysis.

Moving from single-dose DDI studies to multiple-dose studies and observing PK of the victim drug as steady state could provide a situation where NCA would generate more accurate DDI predictions for drugs with a long half-life. Such designs are likely to be costly due to the study length (prolonged dosing to reach steady-state concentrations needed). Further, protracted exposure to a study drug that will persist in the circulation for a long period of time may be undesirable, particularly if the drug has clinically important toxicities. The mentioned disadvantages make single-dose designs and model-based analysis a more appealing option, at least as long as linear PK of the victim drug can be assumed.

The simulation studies show that population PK methods can provide accurate DDI predictions with good precision for drugs with long half-life where NCA methods fail. These simulation studies further demonstrate that metabolite data can be used in the modeling to increase the knowledge gained from the study. Another benefit of the model-based analysis is that it enables a more mechanistic approach wherein the effects of the DDI on primary PK parameter(s) can be tested. Furthermore, the developed model can be used to identify rational dose adjustments for further prospective evaluation in clinical trials when a DDI is judged to be of clinical importance. The three latter advantages are of course present regardless of the victim drug’s half-life.

Population PK methods are mentioned in the regulatory guidance for DDI studies but only as a last resort option to be used for analysis of PK data (often sparsely sampled) collected trials other than dedicated DDI studies (5,6). The same perspective is presented by the organization Pharmaceutical Research and Manufactures of America (PhRMA) (32). This approach can indeed be useful to detect unexpected DDIs or provide evidence in the absence of DDIs. However, population PK has a larger potential than that and we argue for an expanded use of the model-based methods in primary analysis of dedicated DDI studies. It is specifically valuable for drugs with long half-life where NCA analysis may generate biased predictions or demand impractical and expansive study designs, but it also provides additional benefits (making use of metabolite data for improved precision, mechanistic interpretation, dose adjustment simulations) for any victim drug.

## CONCLUSION

Utilizing model-based analysis methods to estimate DDIs is crucial for correct characterization when full concentration-time profiles are not captured. The under-predictions of DDI impact by NCA GMRs can be large enough to result in erroneous and potentially dangerous conclusions and decisions regarding the need for dose adjustment. Model-based analysis of interaction effects is always preferable over NCA for drugs with a long half-life and should be the standard methodology.

## Electronic Supplementary Material

Below is the link to the electronic supplementary material.

ESM 1 (DOCX 60 kb)

ESM 2 (DOCX 37 kb)

ESM 3 (DOCX 48 kb)
