# Supplementary material for: Pharmacokinetic Interactions for Drugs with a Long Half-Life—Evidence for the Need of Model-Based Analysis
Source: AAPS J. 2015 Oct 13;18(1):171–9. doi: 10.1208/s12248-015-9829-2 (PMC4706279; doi:10.1208/s12248-015-9829-2)
Supplement: Supplementary file 1 — (DOCX 60 kb) [file 12248_2015_9829_MOESM1_ESM.docx]

# Supplemental material 1 - Population PK model (1)

**
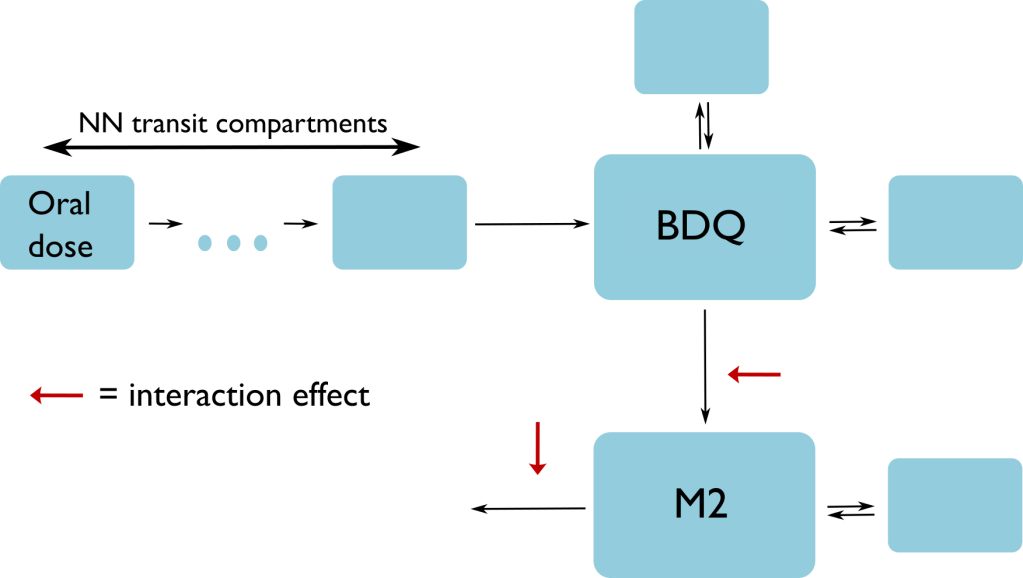
**

| **Parameter estimates with uncertainty** | | | | | | |
| --- | --- | --- | --- | --- | --- | --- |
| **Fixed effects^‡^  (RSE)** |  | **Random effects [CV%] (RSE)** |  |  |  |  |
| **MTT [h]** | 1.31 (12.6%) | **IOV F** | 23.6% (26.9%) |  |  |  |
| **KA [h^-1^]** | 0.128 (8.7%) | **IIV F** | 24.3% (28%) |  |  |  |
| **CL [L h^-1^]** | 2.96 (9.5%) | **IOV MTT** | 55.4% (11%) |  |  |  |
| **V [L]** | 17.3 (18.7%) | **IIV CL** | 23.7% (11.9%) |  |  |  |
| **Q1 [L h^-1^]** | 5.01 (8.3%) | **IIV CLM2** | 29.6%* (31.5%) | 18.8% (23.7%) |  |  |
| **VP1 [L]** | 2870 (15.3%) | **IIV EFVEFF-BDQ** | -69.2%* (13.8%) | -56.1%* (24.1%) | 20.6% (10.8%) |  |
| **Q2 [L h^-1^]** | 4.16 (10.2%) | **IIV EFVEFF-M2** | -29.7%* (36.6%) | -77%* (29.5%) | 75.2%* (17%) | 28.2% (22.1%) |
| **VP2 [L]** | 136 (9%) | **IIV V** | 34.6% (32%) |  |  |  |
| **CLM2 [L h^-1^]** | 12.3 (10.1%) | **IIV Q1** | 18.7% (15%) |  |  |  |
| **VM2 [L]** | 659 (7.2%) | **IIV VM2** | 28.9% (19%) |  |  |  |
| **Q1M2 [L h^-1^]** | 103 (10.5%) | **IIV VP1M2** | 25.9% (39%) |  |  |  |
| **VP1M2 [L]** | 2840 (6%) |  |  |  |  |  |
| **EFVEFF BDQ and M2** | 2.07 (3.6%) | **Prop error TMC** | 23.9% (5.3%) |  |  |  |
| **NN** | 5.21 (20.5%) | **Prop error M2** | 14.9%* (6.1%) | 17.7% (4.8%) |  |  |
| **Error weight TAD <6h** | 1.87 (11%) |  |  |  |  |  |
| **Error weight <BQL** | 3.28 (15%) |  |  |  |  |  |

Abbreviations: MTT = mean transit time, KA = absorption rate constant, F = bioavailability, CL = clearance, V = volume of distribution, Q = intercompartmental clearance, VP = volume of distribution of peripheral compartments, EFVEFF = induction effect of EFV, NN = number of transit compartments, TAD = time after dose, BLQ = below limit of quantification, Prop = proportional, IOV =inter-occasion variability, IIV = inter-individual variability, RSE = relative standard error, CV% = coefficient of variation as percentage
‡ estimated with typical value of F and fraction BDQ metabolized to M2 fixed to one.
* correlation estimated as a covariance.

1. Svensson EM, Aweeka F, Park J-G, Marzan F, Dooley KE, Karlsson MO. Model-Based Estimates of the Effects of Efavirenz on Bedaquiline Pharmacokinetics and Suggested Dose Adjustments for Patients Coinfected with HIV and Tuberculosis. Antimicrob Agents Chemother. 2013 Jan 6;57(6):2780–7.
