# Supplementary material for: Pharmacokinetic Interactions for Drugs with a Long Half-Life—Evidence for the Need of Model-Based Analysis
Source: AAPS J. 2015 Oct 13;18(1):171–9. doi: 10.1208/s12248-015-9829-2 (PMC4706279; doi:10.1208/s12248-015-9829-2)
Supplement: Supplementary file 2 — (DOCX 37 kb) [file 12248_2015_9829_MOESM2_ESM.docx]

# Supplemental material 2 Alternative study designs, estimations without metabolite data


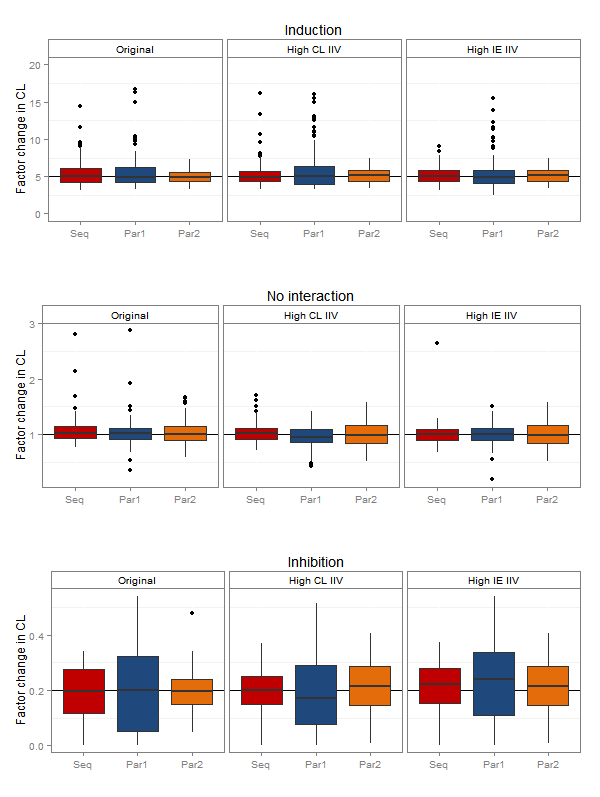


Box-plots of model-based estimation of interaction effect (factor change in CL) excluding metabolite data for the different designs (Seq=sequential, Par1=parallel 1, Par2=parallel 2), the different PK scenarios (original, high CL IIV and high IE IIV) and the different interaction effect scenarios (induction, no interaction and inhibition). A few extreme values are outside of the plot range for the designs sequential and parallel 1 in the induction scenario.
