# Supplementary material for: Pharmacokinetic Interactions for Drugs with a Long Half-Life—Evidence for the Need of Model-Based Analysis
Source: AAPS J. 2015 Oct 13;18(1):171–9. doi: 10.1208/s12248-015-9829-2 (PMC4706279; doi:10.1208/s12248-015-9829-2)
Supplement: Supplementary file 3 — (DOCX 48 kb) [file 12248_2015_9829_MOESM3_ESM.docx]

# Supplemental material 3 Discussion regarding multiple clearance paths for BDQ and M2

### Parameter interpretation

The model is implemented as if BDQ is fully metabolized through the pathway affected by the interaction, but it will correctly predict the changes in BDQ exposure for any fraction of BDQ clearance (CL) being induced or inhibited. However, if the fraction of BDQ metabolized through the induced/inhibited pathway is less than one, the interpretation of the estimated interaction effect (IE_apparent_), i.e. the fractional change in CL with the interaction, does not reflect the change in the pathway associated. Rather, the specific interaction effect on the associated pathway (IE_specific_) can be obtained from:

$${IE}_{specific}=\frac{{IE}_{apparent}+fm-1}{fm}$$

where fm is the fraction metabolized through the pathway in question in the absence of any interaction effect. This fraction will be changed by the interaction and the fraction metabolized through the pathway in question with the interaction (fmi) can be obtained from:

$$fmi=\frac{fm*{IE}_{specific}}{{IE}_{apparent}}$$

For the metabolite, the model is implemented as if all BDQ is forming M2 through the pathway affected by the interactions and all M2 is eliminated via a pathway affected by the interaction. As for BDQ, the model will correctly predict the change in M2 exposure, even if either or both of these assumptions are violated. However, the interpretation of the estimated parameters will change. If M2 is not fully formed or eliminated through the affected pathway, the specific effect on the affected elimination of M2 can be obtained from:

$${IE}_{m,specific} = \frac{{IE}_{specific}*{IE}_{m,apparent}}{{IE}_{apparent}*fmm}-\frac{1}{fmm}+1$$

where IE_m,apparent_ is the estimated interaction effect on M2 CL and fmm is the fraction M2 metabolized through the pathway affected by the interaction in the absence of any interaction effect.

### Relative importance of different elimination pathways

The results from drug-drug interaction studies can be used to draw conclusions regarding the relative importance of different metabolic pathways under certain assumptions. For BDQ and M2 it is known that CYP3A4 is the most important metabolizing enzyme and that BDQ undergoes N-demethylation to form M2 which in term undergoes the same process to form M3 (1). CYP3A4 is the enzyme expected to be affected by lopinavir/ritonavir, efavirenz and rifampicin. It is therefore reasonable to assume that all M2 is formed via the clearance path affected by the interaction. It could also be assumed that potential alternative clearance paths for BDQ, such as fecal excretion, are not affected by the interaction.

From the value of the IE_apperant_, some conclusions can be drawn about the importance of the pathway affected by the interaction for the typical individual. For example, the fact that the IE_apperant_ for the inhibition of BDQ and M2 CL by lopinavir/ritonavir are 0.35 (RSE 9.3%) and 0.58 (RSE 8.7%) (2), respectively, allow the conclusion that the typical fraction BDQ metabolized to M2 through this pathway, fm, cannot be less than 0.65 (95% parametric confidence interval [CI]: 0.59-0.71) and the typical fraction M2 metabolized to M3, fmm, not less than 0.42 (95%CI 0.32-0.52), since a pathway cannot be more than fully inhibited. Given the relationship between the estimated interaction effects on BDQ and M2, the possible combinations of fm and fmm are further limited. Using the estimates from the interaction study with lopinavir/ritonavir and assuming that the direction of the interaction effect on CYP3A4 must be an inhibition, the space of possible combinations of fm and fmm can be confined to the area shown in Figure 1.


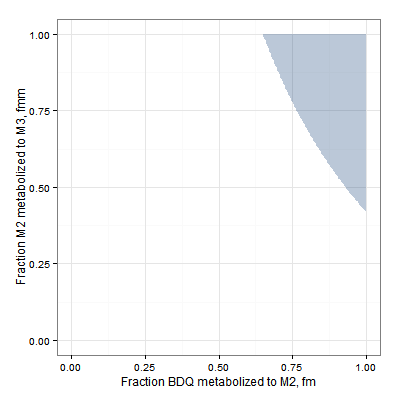


**Figure 1. Space of possible combinations of fraction BDQ metabolized to M2 (fm) and fraction M2 metabolized to M3 (fmm) (blue area) given the estimates of the apparent interaction effects.**

An additional assumption could be that the specific interaction effect on the affected pathway is the same for BDQ and M2. This is plausible in this case since BDQ and M2 are metabolized by the same enzyme affected by the interacting drugs (CYP3A4). Under this assumption the fraction M2 metabolized through the affected pathway is described by:

$$fmm=\left( \frac{{IE}_{specific}*{IE}_{m,apparent}}{{IE}_{apparent}}-1 \right)*\frac{1}{{IE}_{specific}-1}$$

Using the estimates of the apparent interaction effects on BDQ and M2 CL by efavirenz (3) and lopinavir/ritonavir (2) and the uncertainty in the parameter estimates (95% CI), the space of possible combinations of fm and fmm can be further reduced. Possible combinations are those included in the region where the areas from the lopinavir/ritonavir and efavirenz studies are overlapping (darkest region in Figure 2).


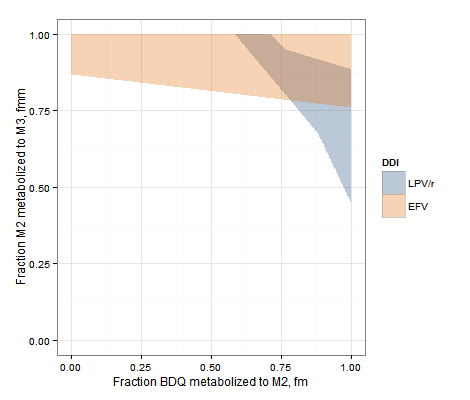


**Figure 2. Space of possible combinations of fraction BDQ metabolized to M2 (fm) and fraction M2 metabolized to M3 (fmm) (dark region where blue and orange overlap) under the assumption that the specific interaction effect is the same for BDQ and M2 and given the estimates of the apparent interaction effects of lopinavir/ritonavir (LPV/r) and efavirenz (EFV) with uncertainty.**

In conclusion, the simplistic parametrization of a parent-metabolite model for drug-drug interactions assuming the fraction metabolized through the clearance pathway affected by the interaction to be one, does not affect the predictions of the impact of the interaction on the exposure. Parameters estimated under this assumption can be transformed to meaningful parameters under other assumptions including multiple clearance paths. The results from drug-drug interaction studies can be used to draw conclusions about the relative importance of different clearance paths given a certain scenario, as demonstrated here for BDQ and M2.

### References

1. van Heeswijk RPG, Dannemann B, Hoetelmans RMW. Bedaquiline: a review of human pharmacokinetics and drug–drug interactions. J Antimicrob Chemother. 2014 Jan 9;69(9):2310–8.

2. Svensson EM, Dooley KE, Karlsson MO. Impact of Lopinavir-Ritonavir or Nevirapine on Bedaquiline Exposures and Potential Implications for Patients with Tuberculosis-HIV Coinfection. Antimicrob Agents Chemother. 2014 Nov;58(11):6406–12.

3. Svensson EM, Aweeka F, Park J-G, Marzan F, Dooley KE, Karlsson MO. Model-Based Estimates of the Effects of Efavirenz on Bedaquiline Pharmacokinetics and Suggested Dose Adjustments for Patients Coinfected with HIV and Tuberculosis. Antimicrob Agents Chemother. 2013 Jan 6;57(6):2780–7.
